# Integrating Physiotherapy in Neuroleptic Malignant Syndrome Management: A Case Report

**DOI:** 10.7759/cureus.62808

**Published:** 2024-06-21

**Authors:** Nikita H Seth, Raghumahanti Raghuveer, Moh'd Irshad Qureshi

**Affiliations:** 1 Neurophysiotherapy, Ravi Nair Physiotherapy College, Datta Meghe Institute of Higher Education and Research, Wardha, IND

**Keywords:** psychiatric disorder, muscular rigidity, clozapine, physiotherapy, neuroleptic malignant syndrome (nms)

## Abstract

Neuroleptic malignant syndrome (NMS) is a rare but potentially fatal condition characterized by hyperthermia, autonomic dysregulation, altered mental status, and muscular rigidity. It typically results from the blockade of dopamine receptors by antipsychotic medications. We present the case of a 70-year-old female who developed NMS after non-compliant use of clozapine. She presented with symptoms including irrelevant talk, breathlessness, and generalized muscle weakness. On examination, she was drowsy with a Glasgow Coma Scale score of 11, tachycardia, tachypnea, and hypertonicity in all limbs. Diagnostic evaluations revealed increased urea and creatinine levels, raised creatine phosphokinase, and metabolic acidosis, which are consistent with NMS. Medical management included the discontinuation of clozapine and the initiation of bromocriptine. The report emphasizes how important physical therapy is to the NMS recovery process. The goals of physical therapy were to improve functional mobility, lessen muscle rigidity, and avoid problems from extended immobility. Kinesthetic stimulation, active cycle breathing methods, soft rocking motions, neural warmth, weight-bearing exercises, and mobility training were all incorporated into the protocol. Significant progress was observed in the patient's degree of consciousness, movement, and oxygen reliance over a two-week period. With the patient eventually managing room air without additional oxygen, the Glasgow Coma Scale score improved, and the ICU Mobility Scale score increased from 1 to 5. This instance emphasizes the need for prompt diagnosis and all-encompassing NMS care, with physiotherapy playing a critical role. Physiotherapy can significantly enhance overall healing, improve respiratory function, and facilitate neuromuscular re-education through tailored therapies. The results indicate that physiotherapy has to be regarded as a crucial component of the multidisciplinary strategy for managing NMS, with the goal of enhancing patient outcomes and quality of life. More studies are required to optimize physiotherapy interventions for NMS patients.

## Introduction

The rare but potentially fatal neuroleptic malignant syndrome (NMS) is characterized by fever, confusion, dysregulation of the autonomic nervous system, and rigidity. In absolute numbers, NMS is a relatively uncommon side effect. According to prevalence estimates, there are between 0.167 and 32.6 instances per 1,000 individuals. Based on a meta-analysis of the epidemiological data found in the literature, an overall estimate of 0.991 cases per thousand persons was obtained [1]. The standard linked agent for NMS is an antipsychotic, and the major trigger is dopamine receptor blockage. Strong conventional neuroleptics with a high potential for side effects, including prochlorperazine, fluphenazine, trifluoperazine, chlorpromazine, and haloperidol, have been linked to NMS the most often. Abrupt withdrawal or dosage reduction of dopaminergic medications, such as levodopa, may potentially cause NMS [2]. There is no evidence that these medications directly affect dopamine. Most experts agree that a marked and sudden decline in central dopaminergic activity brought about by D2 dopamine receptor blockade within the nigrostriatal, hypothalamic, and mesolimbic pathways helps to explain the clinical features of NMS, such as rigidity, hyperthermia, and altered mental status, respectively. The underlying pathophysiologic mechanisms of NMS are complex, with some aspects still up for debate [3].

Various medical conditions mimic NMS presentation, such as heat stroke, central nervous system (CNS) infections, toxic encephalopathies, agitated delirium, status epilepticus, and drug-induced symptoms. Heat stroke differs with abrupt onset, dry skin, hypotension, and limb flaccidity, often caused by neuroleptic medications [4]. CNS infection needs early consideration in NMS cases to prevent treatment delays, with fever, mental status changes, and specific signs such as seizures. NMS diagnosis is complicated by drug-induced syndromes with motor and cognitive features resembling it, caused by various classes of drugs. Serotonin syndrome and malignant hyperthermia share similarities with NMS but can be distinguished by specific symptoms and triggers [5]. NMS requires prompt treatment to avoid severe consequences. Despite the challenges of conducting clinical trials due to the rarity of NMS, treatment guidelines have been established based on case reports and analyses [6]. The initial step in managing NMS involves discontinuing the suspected neuroleptic medication and reinitiating any withdrawn dopaminergic medication. In severe cases, pharmacologic therapy with bromocriptine and dantrolene is often used to shorten the course of the syndrome and reduce mortality. Bromocriptine is given orally to reverse the hypodopaminergic state, while dantrolene can be administered intravenously or orally with close monitoring for hepatoxicity [7].

Neurophysiotherapy is essential for individuals recuperating from NMS. Neurophysiotherapy for NMS aims to reduce muscle rigidity, improve functional mobility, and avoid long-term immobilization-related complications such as contractures and pressure sores [8]. The therapeutic procedures include strengthening, passive and active range-of-motion exercises, and mobility training. Neurophysiotherapy helps patients recover more quickly and thoroughly by addressing these physical limitations, which in turn improves the patient's quality of life and level of independence after NMS [9].

## Case presentation

A 70-year-old female presented to a tertiary care hospital with chief complaints of irrelevant talk and breathlessness for the past four days, along with reduced appetite and generalized muscle weakness for the past eight days. The patient had been in her usual state of health until three months ago when she was diagnosed with a psychiatric disorder and prescribed clozapine (an antipsychotic). However, she was non-compliant with her medications. Three days prior to admission, she visited the respiratory medicine department with similar complaints of breathlessness and was advised to take albuterol. After the first dose, she began exhibiting irrelevant talk and altered sensorium, prompting her to report to the hospital. Additionally, the patient has a known history of a psychiatric disorder for the past three months and has been on clozapine, ischemic heart disease managed with Ecospirin 75 mg, hypertension for three years managed with telmisartan (OD), and bronchial asthma.

On examination, the patient was drowsy with a Glasgow Coma Scale score of 11 (eye-opening: 3, verbal response: 3, and motor response: 5). The body build was ectomorphic. The patient had tachycardia and tachypnea with a saturation of 97% and 3 L of oxygen via face mask. Pallor was present, and accessory muscle was used. During the sensory examination, the patient responds to painful sensations by making facial gestures. Other sensations could not be assessed as the patient was drowsy. On motor examination, a tone grading scale of 3+ indicates the presence of hypertonicity in the bilateral upper and lower limbs, along with trunkal rigidity. There was bi-compartmental resistance to passive movement. The type of rigidity was a lead pipe (constant resistance throughout the range of motion). Hyporeflexia was observed. Postural tremors were also present, and tendoachilles tightness was more present on the right side than on the left. Table 1 depicts the examination findings for deep tendon reflexes.

**Table 1:** Examination findings for deep tendon reflexes + represents diminished reflex

	Bicep Jerk	Tricep Jerk	Supinator Jerk	Knee Jerk	Ankle Jerk	Plantar Response
Right	+	+	+	Absent	+	Flexor
Left	+	+	+	+	+	Flexor

Investigations

**Figure 1:**
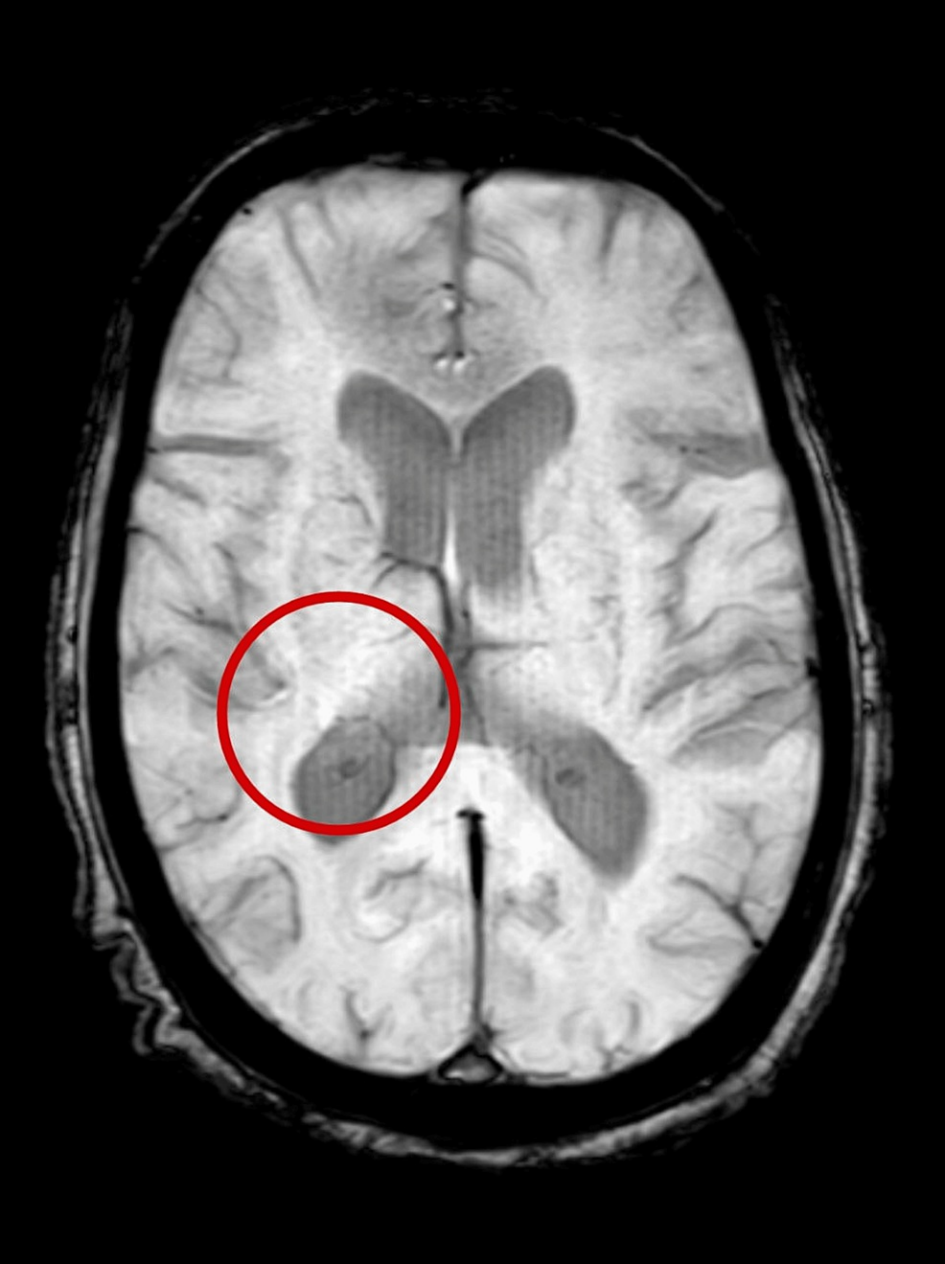
Magnetic resonance imaging findings The red circle indicates small vessel ischemic changes involving bilateral frontoparietal periventricular white matter with Fazekas grade 1, along with age-related cerebral atrophy.

A kidney function test was performed, which revealed an increased urea level (103 mg/dL) and creatinine level (2.0 mg/dL). Arterial blood gas analysis was done, which revealed compensated metabolic acidosis. The creatine phosphokinase (600 IU/L) level was raised, suggestive of muscle damage. According to the Diagnostic and Statistical Manual of Mental Disorder (DSM), severe muscle rigidity was present, associated with the use of neuroleptic medications and tremors, change of consciousness level, tachycardia, and increased level of creatinine phosphokinase (CPK). Magnetic resonance imaging (MRI) revealed small vessel ischemic changes involving bilateral frontoparietal periventricular white matter with Fazekas grade 1, along with age-related cerebral atrophy. Figure 1 depicts MRI findings.

Physiotherapy interventions

The medical management involved initiating treatment with bromocriptine 2.5 mg, which is a neuroleptic medication. In addition, a two-week physiotherapy intervention was given, which was focused on reducing rigidity, improving functional mobility, reducing work breathing, preventing secondary complications, and improving overall well-being. Table 2 includes a concise description of the physiotherapy rehabilitation protocol.

**Table 2:** Brief physiotherapy rehabilitation protocol

Goals	Intervention	Rationale
To improve the level of consciousness	Kinesthetic stimulation (15-minute session)	Faster movements to facilitate arousal. Mobility activity promotes body and positional awareness
To reduce the work of breathing	Active cycle of breathing technique and purse lip breathing exercise	Opening up of interbronchiolar channels of Martin and alveolar pores of Kohns
To normalize the muscle tone	Gentle rocking movements and neural warmth rotation of the extremities and trunk	It helps to achieve relaxation
To reduce tremors	Weight-bearing exercises, behavioral relaxation therapy	A cognitive approach to elicit a relaxation response in stressful situations that counteracts the stress response
To improve scapula and upper limb mobility	Reach-out exercises in sitting	Helps to improve scapular mobility and targets functional performance
To gain independence in bed mobility	Bed mobility training	In order to prevent complications secondary to immobilization

In order to minimize problems such as deep vein thrombosis, pressure sores, and contractures that are linked to extended immobility, physiotherapy plays a supportive role in the management of non-motor symptoms. As the patient's condition stabilized, physiotherapists worked on respiratory therapy to prevent pneumonia, progressive mobilization to restore functional independence, and passive and active range-of-motion exercises to maintain joint mobility. A customized strategy and close observation were the key elements during the recovery period due to the patient's general frailty and changing clinical state.

**Figure 2:**
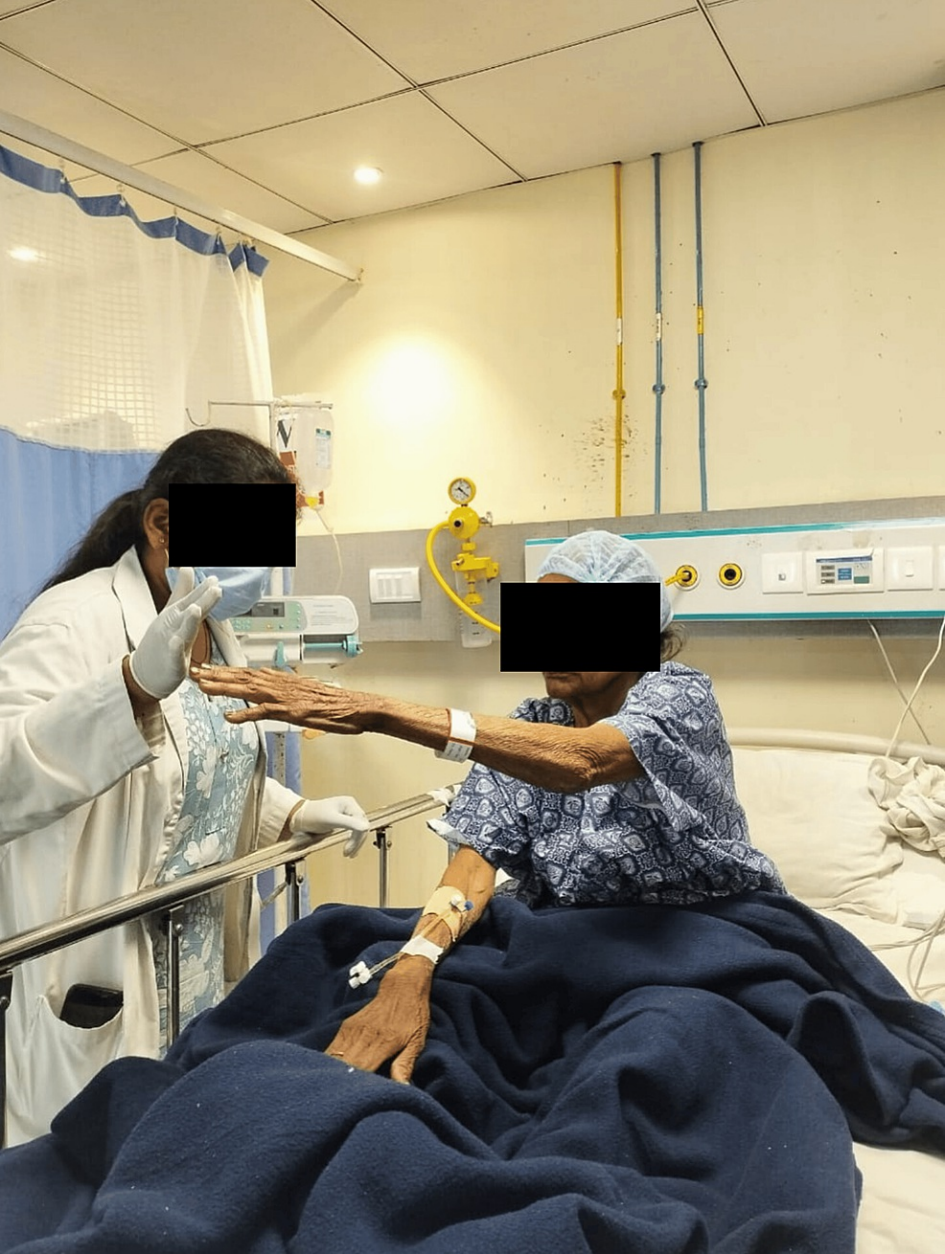
Reach-outs in sitting

Figure 2 shows the patient performing the reach-out activity in a sitting position.

**Figure 3:**
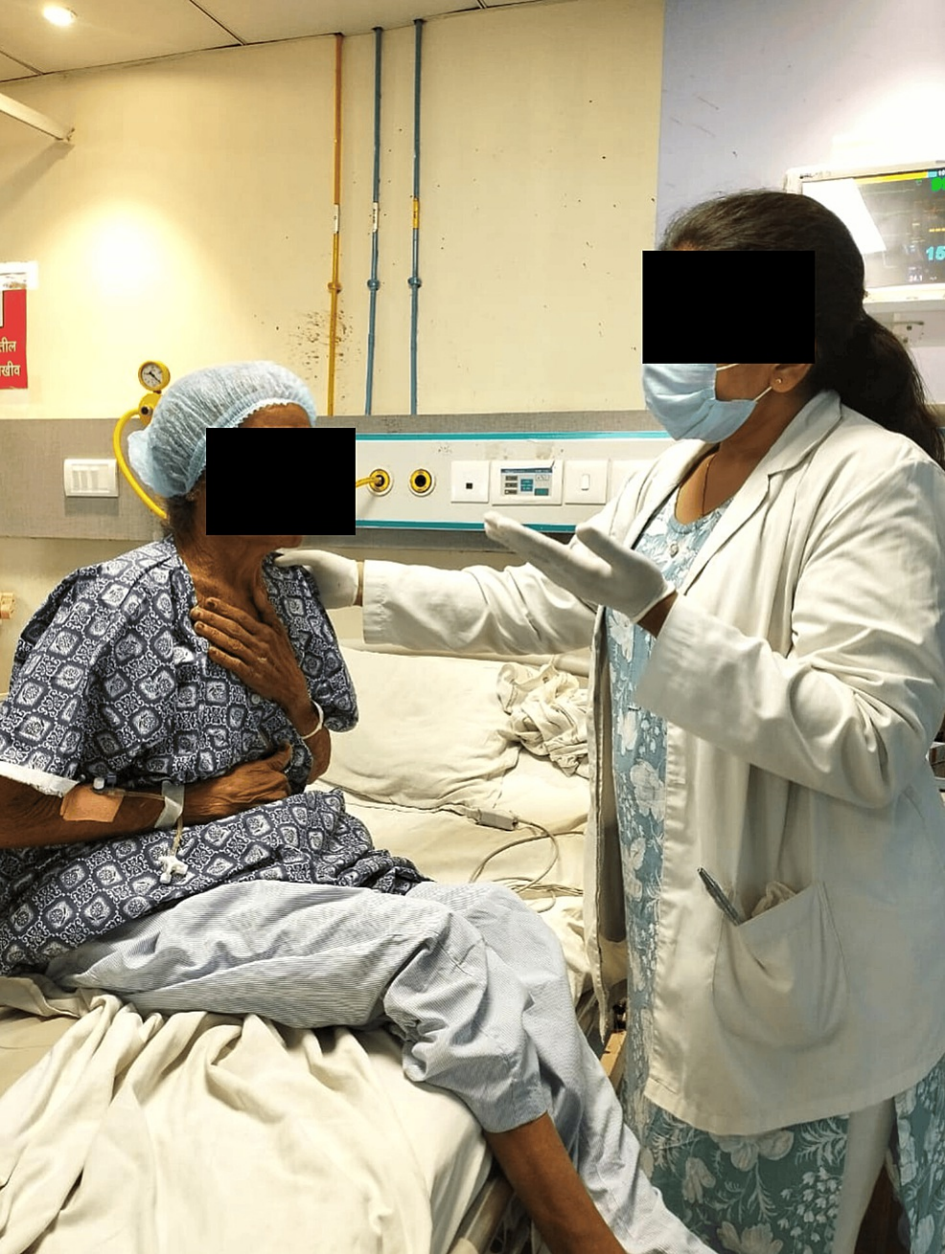
The patient performing breathing exercises

Figure 3 shows the patient performing breathing exercises.

Outcome measures

Three outcome measures were assessed before the treatment, and the same outcomes were assessed after two weeks (14th day). There was a significant improvement in the intensive care unit (ICU) mobility scale score and improvement in the Glasgow Coma Scale score, suggestive of improvement in the level of consciousness, as mentioned in Table 3.

**Table 3:** Outcome measures used in pre- and post-rehabilitation ICU: intensive care unit; E: eye-opening; V: verbal response; M: motor response

Outcome Measures	Pre-treatment (Day 1)	Post-treatment (Day 14)
Glasgow Coma Scale	E3V3M3	E4V4M6
ICU mobility scale score	1	5
Oxygen requirement	3 L of O_2_ via face mask	On room air

## Discussion

Although cases of NMS have been documented following the use of both low-potency first-generation and second-generation antipsychotic medicines, NMS is primarily observed with the use of high-potency first-generation antipsychotic treatments, such as haloperidol and fluphenazine [10]. NMS has also been linked to selective antiemetic medications, including levosulpiride, promethazine, and metoclopramide. The patient showed clear indications of NMS following a known use of clozapine for three months. A constellation of symptoms, such as arrhythmias, hyperpyrexia, labile blood pressure, and muscle cramps and rigidity, typically precede NMS. D2-receptor blockage is hypothesized to be the origin of NMS muscular rigidity, which can present itself in a variety of forms, such as oculogyric crisis and choreiform movements. However, development and degradation frequently happen quickly. Following are hematological and biochemical alterations, including leukocytosis, increased serum CPK as a result of hyperkinesia and rhabdomyolysis, and metabolic acidosis [11].

In terms of clinical presentation, NMS must be differentiated from its mimics, which include malignant hyperthermia, toxic encephalopathy, and serotonin syndrome encephalitis. All of these conditions present with a similar constellation of symptoms. Before starting the proper course of treatment and differentiating NMS from other differentials, a thorough and comprehensive medication history must be obtained. The offending agent must be stopped as soon as NMS is detected. It is essential to maintain a euvolemia state and lower CPK levels to ideal levels [12]. Most NMS cases go well within two weeks (the average being 7-11 days). Over the past few decades, NMS mortality rates have exceeded 30%. Currently, the mortality rate is 5%-20%. There is a very high risk of lifelong brain impairment if there has been acute hypoxia or noticeably prolonged higher temperatures. The average duration for NMS cases to resolve is typically 7-11 days, with the majority of cases falling within this timeframe. There is a paucity of research on the role of physiotherapy in NMS [13].

Integrating physiotherapy interventions along with medical management, including passive and active range-of-motion exercises, respiratory muscle training, and functional mobility training, helps to mitigate complications such as muscle rigidity, contractures, and respiratory dysfunction [14]. By enhancing mobility, reducing the risk of deep vein thrombosis, and promoting overall physical function, physiotherapy has significantly improved outcomes, aiding in faster recovery and reducing the length of hospital stay for patients with NMS [15].

## Conclusions

This case study highlights the significance of prompt diagnosis and thorough treatment for NMS, a potentially fatal illness characterized by severe symptoms. The patient's rehabilitation was greatly aided by the incorporation of physiotherapy, which also reduced oxygen dependency and increased movement and alertness. Physiotherapy promoted neuromuscular re-education, improved respiratory function, and avoided problems associated with immobility by means of focused interventions such as respiratory exercises, progressive mobilization, and passive and active range-of-motion exercises. An inherent limitation of this study is its implementation on a single patient, making it difficult to attribute improvements solely to physiotherapy. Additionally, the short follow-up period limits the assessment of long-term outcomes.
